# A Model of Ant Route Navigation Driven by Scene Familiarity

**DOI:** 10.1371/journal.pcbi.1002336

**Published:** 2012-01-05

**Authors:** Bart Baddeley, Paul Graham, Philip Husbands, Andrew Philippides

**Affiliations:** 1 Centre for Computational Neuroscience and Robotics, Department of Informatics, University of Sussex, Brighton, United Kingdom; 2 Centre for Computational Neuroscience and Robotics, School of Life Sciences, University of Sussex, Brighton, United Kingdom; University of Bielefeld, Germany

## Abstract

In this paper we propose a model of visually guided route navigation in ants that captures the known properties of real behaviour whilst retaining mechanistic simplicity and thus biological plausibility. For an ant, the coupling of movement and viewing direction means that a familiar view specifies a familiar direction of movement. Since the views experienced along a habitual route will be more familiar, route navigation can be re-cast as a search for familiar views. This search can be performed with a simple scanning routine, a behaviour that ants have been observed to perform. We test this proposed route navigation strategy in simulation, by learning a series of routes through visually cluttered environments consisting of objects that are only distinguishable as silhouettes against the sky. In the first instance we determine view familiarity by exhaustive comparison with the set of views experienced during training. In further experiments we train an artificial neural network to perform familiarity discrimination using the training views. Our results indicate that, not only is the approach successful, but also that the routes that are learnt show many of the characteristics of the routes of desert ants. As such, we believe the model represents the only detailed and complete model of insect route guidance to date. What is more, the model provides a general demonstration that visually guided routes can be produced with parsimonious mechanisms that do not specify when or what to learn, nor separate routes into sequences of waypoints.

## Author Summary

The interest in insect navigation from diverse disciplines such as psychology and engineering is to a large extent because performance is achieved with such limited brain power. Desert ants are particularly impressive navigators, able to rapidly learn long, visually guided foraging routes. Their elegant behaviours provide inspiration to biomimetic engineers and for psychologists demonstrate the minimal mechanistic requirements for complex spatial behaviours. In this spirit, we have developed a parsimonious model of route navigation that captures many of the known properties of ants routes. Our model uses a neural network trained with the visual scenes experienced along a route to assess the familiarity of any view. Subsequent route navigation involves a simple behavioural routine, in which the simulated ant scans the world and moves in the most familiar direction, as determined by the network. The algorithm exhibits both place-search and route navigation using the same mechanism. Crucially, in our model it is not necessary to specify when or what to learn, nor separate routes into sequences of waypoints; thereby providing proof of concept that route navigation can be achieved without these elements. As such, we believe it represents the only detailed and complete model of insect route guidance to date.

## Introduction

The impressive ability of social insects to learn long foraging routes guided by visual information [1]–[8] provides proof that robust spatial behaviour can be produced with limited neural resources [9]–[11]. As such, social insects have become an important model system for understanding the minimal cognitive requirements for navigation [12]. This is a goal shared by biomimetic engineers and those studying animal cognition using a bottom-up approach to the understanding of natural intelligence [13].

In this field, computational models have proved useful as proof of concept [14], [15] that a particular sensori-motor strategy [16] or memory organisation [17] can account for observed behaviour. Such models of visual navigation that have been successful in replicating place homing are dominated by snapshot-type models; where a single view of the world as memorized from the goal location is compared to the current view in order to drive a search for the goal [16], [18]–[26]. Snapshot approaches only allow for navigation in the immediate vicinity of the goal however, and do not achieve robust route navigation over longer distances [27], [28]. Here we present a parsimonious model of visually guided route learning that addresses this issue. By utilising the interaction of sensori-motor constraints and observed innate behaviours we show that it is possible to produce robust behaviour using a learnt holistic representation of a route. Furthermore, we show that the model captures the known properties of route navigation in desert ants. These include the ability to learn a route after a single training run and the ability to learn multiple idiosyncratic routes to a single goal. Importantly, navigation is independent of odometric or compass information, does not specify when or what to learn, nor separate the routes into sequences of waypoints, so providing proof of concept that route navigation can be achieved without these elements. The algorithm also exhibits both place-search and route navigation with the same mechanism.

### Navigation in ants

Individual ant foragers show remarkable navigational ability, shuttling long distances between profitable foraging areas and their nest. Despite low resolution vision and the availability of odometric information, many ant species preferentially guide their foraging routes using learnt visual information [2], [29]–[31]. The robust extraction and learning of the visual information required for route guidance is a product of the interactions between innate behaviours and learning [12], [32]. We highlight these interplays by sketching out the career of an individual forager.

Upon first leaving the nest, a new forager performs a series of short *learning walks* where a carefully orchestrated series of loops and turns allow her to inspect the visual surroundings from close to the nest entrance [33]–[35]. The knowledge gained during these special manoeuvres means she will be able to use visual information to pin-point the nest entrance after future foraging trips. When she finally leaves the vicinity of the nest she is safely connected to it because of her path integration (PI) system [12], [36]. In order to perform path integration, odometry and compass information are continuously combined such that at all times during a foraging journey the ant has the direction and distance information required to take an approximately direct path home. However, PI is subject to cumulative error and cannot take account of passive displacements, such as by a gust of wind. To mitigate these risks and ensure robust navigation, ants therefore learn the visual information required to guide routes between the nest and their foraging grounds [Reviews: [12], [37]]. During the early stages of learning the ants are reliant on their PI system for homing. However, as they become more experienced they come to rely more and more on visual information for route guidance [31]. The use of PI also provides consistent route shapes thereby facilitating and simplifying the learning of appropriate visual information [32], [38].

Extensive behavioural experiments over many years have led to a knowledge base of properties and behavioural signatures of visually guided navigation in ants that can be summarised as follows:

1. Ants can use visual information to guide routes between their nest and a stable food site [1], [3], [8], [39].
2. Routes are idiosyncratic, so individual ants will adopt and remain faithful to unique routes [1]–[3], [8].
3. Routes have a distinct polarity; knowledge of a nest-food route does not imply knowledge of a food-nest route [4], [9].
4. Route knowledge defines a *visual corridor* as opposed to a narrow ridge, so the overall shapes of routes are stable but ants do not have to recapitulate them with high precision [1], [3], [9], [39].
5. The visual knowledge used to define routes can be used independently of any odometric information that the ant may possess [1]–[3], [31], [40], [41].
6. Route following is not dependent on learning a strict sequence of actions. The knowledge needed to guide a route can be accessed out of the usual sequence [1], [3].
7. Ants can use learnt visual information to drive a search for their nest entrance [2], [42]–[44].
8. The visual information required to follow a route can be learnt very rapidly; however performance becomes more stable with experience [34], [43], [45].
9. Individual ants can learn multiple routes to the same destination [6].

### Models of visually-guided navigation in insects

Computational models of visual navigation in insects followed experimental findings where ants [42] and bees [16] had been shown to guide their return to a goal-location by matching retinotopic information as remembered from the goal. With their seminal snapshot model, Cartwright and Collett [16] showed that within a certain catchment area [46] subsequent search for a goal location can be driven by a comparison of the current view of the world and a view stored at that goal. This has inspired roboticists and biologists to develop homing models [19]–[26] where a single retinotopic view is used to get back to a location.

Snapshot style models represent elegant, but abstract, sensori-motor strategies for navigation yet there are two major directions where such models need developing. Firstly, although snapshot models are very useful for understanding the information that is available in a visual scene [21], [47], to fully understand visual navigation we must consider the constraints imposed by a particular motor system and means of locomotion. Secondly, we need to understand how visual knowledge can be applied to the guidance of longer distance journeys and not just to the pin-pointing of a single goal location.

### Understanding sensori-motor interactions

A significant component to any view-based homing algorithm is the sensori-motor interaction. The original snapshot model was developed following extensive experiments with bees. In the final stages of locating an inconspicuous goal, bees and wasps are able to fix the orientation of their body axis, perhaps using compass information, and then translate in any direction [48]–[50]. Inspired by this, the original snapshot model relies on stored views and current views being aligned to an external frame of reference before a matching procedure is used to determine a homing direction [16]. This represents a significant challenge for ants, and also for bees and wasps when flying rapidly over longer distances, where translation is predominantly in the direction of the body axis. In the context of our proposed model, however, the tight coupling of sensation and action is used to simplify the problem of learning a route. For an ant with fixed eyes and a relatively immobile head a given view implicitly defines a direction of movement and therefore an action to take. This suggests the following approach:

#### Our approach

A panoramic image can be used as a *Visual Compass*; the difference between a goal image and rotated images from nearby locations is minimised when the rotated images are at the orientation of the original [21], [47], [51]. Therefore, rather than tagging remembered images with an orientation, or rotating into a particular orientation during learning and recall, we use a similar mechanism to a visual compass to search for familiar viewing directions. The fact that ants are moving, and therefore facing, in the overall route direction most of the time during learning means that these familiar viewing directions implicitly define the movement directions required to stay on the route. It is therefore sufficient to learn all of the views exactly as they are experienced. The problem of navigation is then re-framed in terms of a rotational search for the views associated with a route. By visually scanning the environment and moving in the direction that is most similar to the views encountered during learning an ant should be able to reliably retrace her route.

Note that this process associates the current view not with a particular place but instead with a particular action, that is, “what should I do?” not “where am I?”. In addition, it means that compass information is not necessary during either learning or recall [52].

### Using views for route guidance

Given the success of snapshot-type models in place-homing, it is natural to assume that navigation over larger scales, that is, along routes, could be achieved by internalizing a series of stored views linked together as a sequence. Route behaviour in this framework would entail homing from one stored view to the next in a fixed sequence. While it has been shown that the catchment areas of individual snapshots can be quite large [21]–[47], [53], attempts to model route navigation using linked view-based homing have shown it to be a nontrivial problem which requires the agent to both robustly determine at which point a waypoint should be set during route construction and when a waypoint has been reached during navigation [19], [27], [28]. Essentially, for robust route navigation using a sequence of snapshots, an agent needs place recognition to determine where along the route it is [54]. Here we propose a different model that develops and refines ideas that have been recently put forward as an alternative to such a scheme [55]. Instead of defining routes in terms of discrete waypoints all views experienced during training are used to learn a holistic route representation.

#### Our approach

An artificial neural network is first trained using the views experienced during a return to the nest. During subsequent navigation the network is used to estimate whether a given view has been experienced before. A behavioural routine facilitates route following by scanning the world and moving in the direction that is most familiar and therefore deemed most likely to be part of the route.

We feel that this approach has two major benefits. Firstly, we do not attempt to learn in detail specific views along the route, but instead use all of the views to determine a measure of familiarity. In this way our approach provides a compact way of storing the visual information required to follow routes that is also open-ended in that new information can be incorporated at any time in the future. As a corollary, the agent does not need to decide when or which views to learn. Secondly, the agent does not need to determine where along the route it is. By performing visual scans of the world from the current location and moving in the direction that appears most familiar we obviate the need to determine a sequence of views that must be experienced in the correct order.

Both desert ants and wood ants perform scanning behaviours that support this approach. When released in an unexpected but familiar place the desert ant *Melophorus bagoti* scans the environment by turning rapidly on the spot [A. Wystrach and P. Graham, personal observation]. More than one scan may be performed with short straight runs of a few centimetres separating them before the ant finally sets off in a seemingly purposeful manner. The desert ant *Cataglyphis bombycina* has also been reported to perform a similar scanning behaviour during foraging runs [56], [57]. Wood ants exhibit a second form of scanning behaviour; instead of walking in a straight line, they tend to take a sinuous path [58] which has the effect of producing scans of the world centred on the overall direction of movement.

### Preview

We test our proposed route navigation strategy in simulation, by learning a series of routes through visually cluttered environments consisting of objects that are only distinguishable as silhouettes against the sky. This represents a challenging task due to the paucity of information and the potential for visual aliasing, whereby two locations appear similar enough so as to be indistinguishable. Our results indicate that, not only is the approach successful, but also that the routes that are learnt show many of the features that characterise the routes of desert ants.

## Results

### Navigating with a perfect memory

Our navigation algorithm consists of two phases. The ant first traverses the route using a combination of PI and obstacle avoidance (as specified in the Materials and Methods) during which the views used to learn the route are experienced. Subsequently, the ant navigates by visually scanning the world and moving in the direction which is deemed most familiar. In the later experiments, the route is learnt by a neural network and the familiarity of each view is the output of the trained network. However, to show the utility of the proposed scanning routine, without the added complication of learning a familiarity metric, we first explored the performance of a system with perfect memory. This was implemented by storing views experienced every 4 cm along a training route and using these to determine view familiarity directly. Following Zeil et al. [21] we calculate the sum squared difference in pixel intensities between rotated versions of the current view and each stored view. The minimum across all stored images and all viewing directions experienced during a 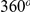 scan of the world from the current location is deemed the most familiar view for that location and a 10 cm step is taken in the viewing direction associated with this minimum.

**Figure 1:**
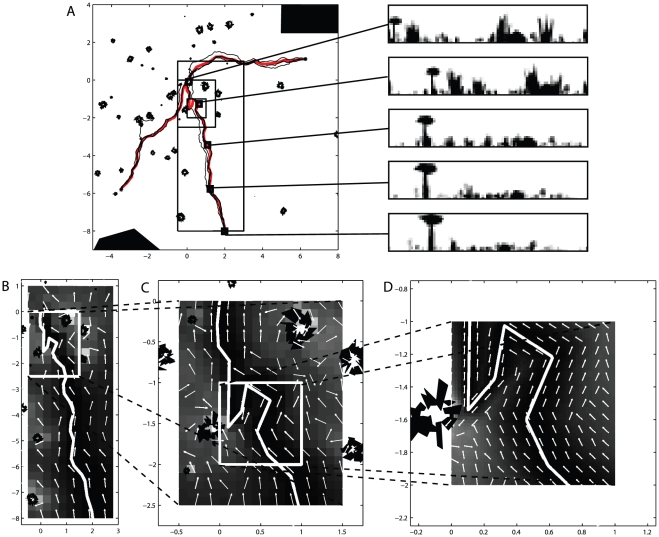
Navigating with a perfect memory. A) Three separate routes (red lines) learned in an environment containing both small and large objects. For each of the three routes, that consisted of between 700 and 980 views taken every 1 cm, we show three recapitulations (black lines). During route recapitulations the headings at each step were subject to normally distributed noise with a standard deviation of 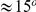. The panels to the right of the main figure show example views from points (indicated by squares) along the training route. B,C,D) Various sections of the middle route at a variety of different scales. The figures show the result of running the navigation algorithm at each point within a grid and indicate what action would be taken by an agent placed at that location. The white line indicates the training path and the white arrows indicate the directions that would be chosen from those locations. The underlying pseudocolour plot indicates the quality of the best match to the stored views for each position, with darker hues indicating a better match.

Figure 1 shows that by storing the views along a training path and using these to drive a subsequent recapitulation of the route, robust behaviour is achievable. We used our algorithm to learn three routes through an environment containing both small and large objects randomly distributed across the environment. Three subsequent navigation paths were attempted for each route. Of the nine paths, all but one successfully return to the nest location, with the one failure caused by the simulated ant being drawn out of the stable *route corridor* by the presence of a tussock that dominates the visual field and causes visual aliasing. Despite the noise added to the movements during the recapitulation, paths are idiosyncratic though inexact. Within a corridor centred on the original route both a good match and a sensible heading are recovered that will, in general, drive the simulated ant towards the goal (Figures 1B–D). Outside of this route corridor the best match becomes poorer and, particularly within areas containing a high degree of visual clutter (i.e. within a group of tussocks) the proposed direction of movement less reliably points towards the goal. This is seen most clearly in panel C where, very close to tussocks, a significant proportion of the homeward directions determined by the algorithm (white arrows in Figure 1B–D) point away from the goal location. Often these erroneous signals direct movements back into the route corridor, although this is clearly a matter of chance.

The routes that are generated show a distinct polarity meaning that they can only be traversed in a single direction as is evidenced by the coherence of the homeward directions (arrows in Figure 1B–D). Importantly, the actions that result from following this strategy are not tied to a coordinate system and are therefore completely independent from the PI system that provided the initial scaffold for learning. In addition, the resulting routes are not dependent on a chained sequence of actions; appropriate actions are taken at any location along the route corridor independent of how that location was reached.

### Adding learning walks

**Figure 2:**
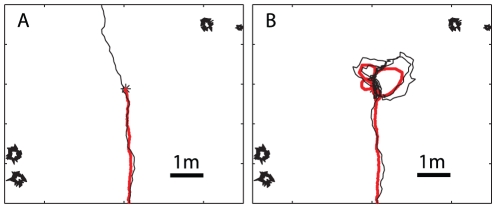
Including learning walks prevents return paths from overshooting the goal. A) Without a learning walk the simulated ant overshoots and carries on in the direction it was heading as it approached the nest location. B) By including the views experienced during a learning walk the simulated ant, instead of overshooting, gets repeatedly drawn back to the location of the nest. Red lines training paths, black lines recapitulations.

One potential problem with this navigational strategy is that if the simulated ant overshoots the goal it will, in general, carry on heading in the same direction and move further and further away from the goal location (Figure 2A). This is because there are no training views that point back towards the nest once it has been passed. This problem can be mitigated by including an exploratory learning walk during the training phase, a behaviour seen in many species of ants [33]–[35]. These initial paths take the form of a series of loops centred on the nest as can be seen in Figure 2B which shows the learning walk of a *Melophorus bagoti* worker taken from a paper by Muser et al. [33]. Essentially, this process means that in the region around the nest there will always be some views stored which are oriented back towards the nest.

To explore the possible effects of these initial short learning walks, the views experienced along them were added to the set of inbound views used for route learning. Figure 2 shows the end section of a route navigated after training with and without a learning walk. In these tests the simulation was not stopped when the simulated ant reached the nest location, analogous to blocking the nest entrance in a behavioural experiment. With the addition of a learning walk (Figure 2B), as the simulated ant passes the nest, rather than the best match being from the training route and oriented upwards (as in Figure 2A), the best match comes from the learning walk. The simulated ant is drawn into the loop of the learning walk it first encounters, leading to the looped paths seen in Figure 2B. Close to the nest, the density of points from the learning walk increases and there are multiple views from nearby locations oriented in a variety of directions. The best match at subsequent points will then likely be from different learning walk loops and so the ant stops following a single loop and enters more of a search-type path around the nest. Thus, our algorithm demonstrates both route following and nest search with the same mechanism.

### Summary

Here we have shown that by storing and using panoramic views as they were experienced and aligned during training, we can achieve visually guided route navigation through a scanning routine and without recourse to a compass. The model is of particular interest since the resulting paths show remarkable similarities to many of the features that we observe in the routes of ants. Specifically, independence from the PI system that is assumed to scaffold the original learning; distinct polarity of routes; formation of a route corridor; and procedural rules that can be accessed out of sequence. By including a learning walk we can also get visually driven search for the nest location from the same mechanism. This algorithm demonstrates the efficacy of using a simple scanning behaviour as a strategy for seeking familiar views. However, the algorithm relies on the unrealistic assumption of a perfect memory of views experienced along the training route. We next investigate a more realistic encoding of the visual information required for navigation by training an artificial neural network using the views experienced along a return journey and a learning walk.

### Familiarity and Infomax

Having shown that the proposed scanning routine can produce *ant-like* paths, we next addressed the problem of learning a familiarity metric to use in place of a perfect memory system. Instead of storing all of the views experienced on a training route, the views were used to train a two-layered artificial neural network to perform familiarity discrimination using an Infomax learning rule [59]. Each view was presented to the network in the order in which it was collected and then discarded. This means that the memory load does not scale with the length of route but remains constant. Once trained, the network takes a panoramic image as input and outputs a familiarity measure indicating the likelihood of the view from that location and orientation being part of the learnt route. The trained network was then used in conjunction with the scanning routine to drive route navigation by presenting the rotated views to the network, and choosing the most familiar direction as the direction in which to navigate. The only difference in the behavioural routine was that the scanning range was reduced from 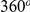 to a slightly more realistic 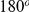 scan centred on the direction of travel from the previous timestep.

**Figure 3:**
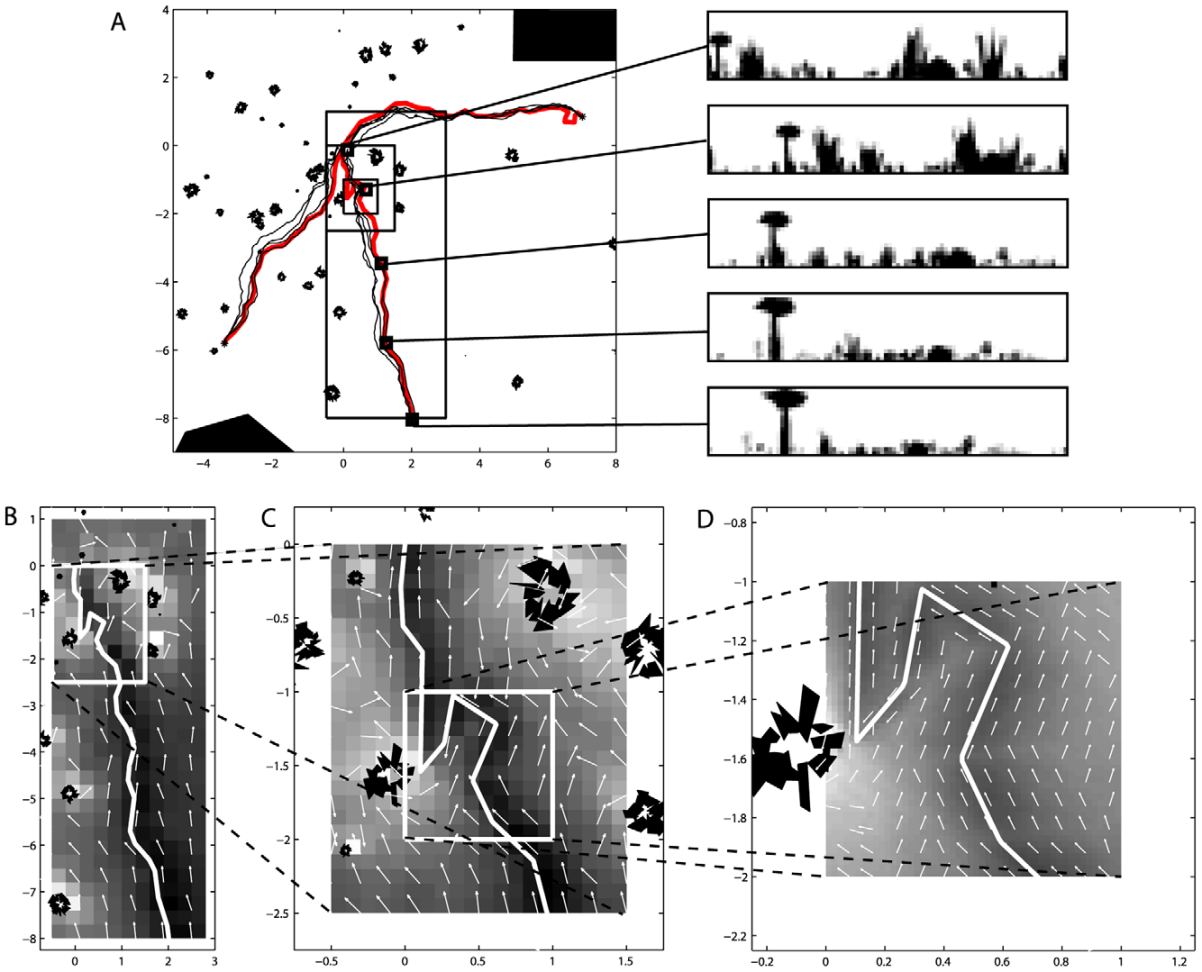
Navigating using a trained artificial neural network to assess scene familiarity. A) Successful return paths for three different routes. The panels to the right of the main figure show example views from points (indicated by squares) along the training route. B,C,D) Various sections of the middle route at a variety of different scales. The pseudocolour plots indicate the familiarity of the best view as was output from the trained network, with darker hues indicating increased familiarity. Conventions as in Figure 1.

In a first experiment using this approach we employed the Infomax system to learn the same three training paths as in the previous experiment using a perfect memory. As Figure 3 shows, in this instance all returns were completed successfully. We do not believe this indicates that the approach is more robust than the perfect memory system but simply that the noise added to the system did not happen to nudge the agent into an area of the environment where visual aliasing would occur. In other ways the results of this experiment are very similar to the results obtained using a perfect memory. As these routes were learned using a single exposure to each of the training views, we are thus able to fulfil another of the desiderata for ant-like visual mediated route navigation: that routes can be learnt rapidly, in this case following a single trial.

To show that learning was not environment specific we conducted further simulations. Environments with varying densities of tussocks were randomly generated and a simple algorithm that performed path integration with obstacle avoidance was used to generate paths through them. In all of the environments we provided a distant horizon consisting of bush-like and tree-like objects as would be present in the natural environment of *Melophorus bagoti* [33]. In these experiments we also included a simplified learning walk at the start of training to prevent the simulated ant overshooting the goal.

**Figure 4:**
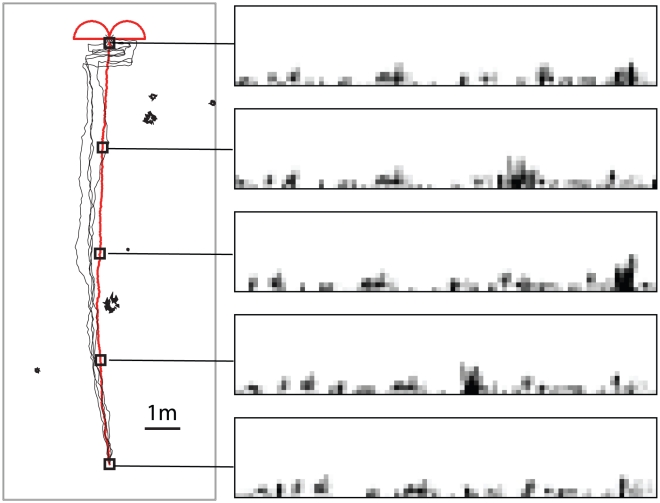
Navigational performance in a sparse environment with a tussock density of 0.05 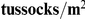. The left panel shows the training (red) and test (black) paths for a 12 m route. The right panel shows example views from points (indicated by squares) from the training route. The combined learning walk and training route consisted of 520 views that were used to train the network.

We first examined a low tussock density environment compared to the environment used previously. Performance was good, although the lack of nearby objects resulted in less consistent paths (Figure 4). Example views taken from the training route (Figure 4, right) show how the panorama of distant objects provide a stable frame of reference throughout the route. Despite the sparse visual information in this environment, the distant objects help to keep the return paths heading in the right direction. The effect that the structure of the learning walk has on the return paths can be clearly seen near the goal location. As the simulated ant nears the goal it gets drawn into a series of left and right sweeps that reflect the left and right inbound loops of the learning walk and are analogous to an ant's search for its often inconspicuous nest entrance.

**Figure 5:**
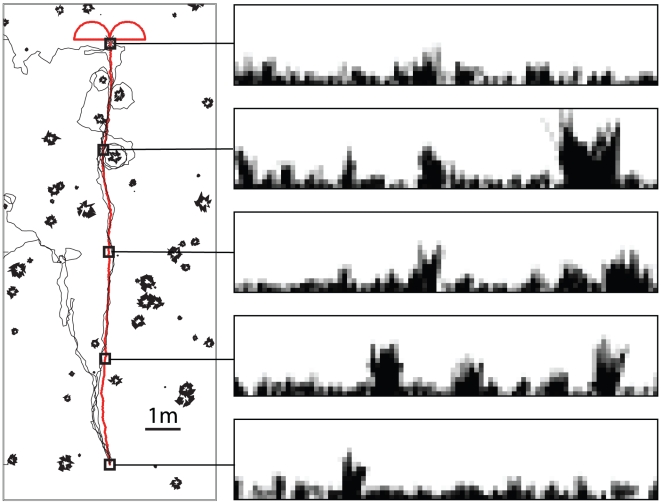
Navigational performance in a cluttered environment with a tussock density of 0.75 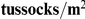. The left panel shows the training (red) and test (black) paths for a 12 m route, squares indicate points where example views from the training run (right panel) are taken from. The combined learning walk and training route consisted of 520 views that were used to train the network.

We next used an environment with a more dense set of tussocks (Figure 5). In this more densely tussocked world the distant panorama is no longer visible at all points along the route. This clearly makes route learning more difficult as is evidenced by the failures in three of the four runs. Because noise is added to the simulated ant's heading during route recapitulation the simulated ant may stray into previously unexperienced parts of the environment which, even a short distance away from the learnt route, can look very different in this cluttered world. Two attempts fail early when noise added to the heading leads the simulated ant to go to the left of a small tussock taking it into a part of the world with which it is not familiar. The other two returns do reasonably well. They do show some circling of tussocks, driven by training views where the path goes very close to a tussock and dominates the visual field, however both paths make it very close to the nest. This is a challenging environment in which to navigate and was picked to be at the limit of the algorithm's learning power following just one training run; other runs using a similar density of tussocks were more successful. Performance also improved if we removed or reduced the noise that was added to the direction of movement at each timestep. Of course, ordinarily ants would incorporate knowledge from several foraging trips during which time their performance becomes more stable and robust. We investigate this in the next section.

### Route performance improves with experience

Performance of our algorithm was often quite reliable following a single training run but there were still failures (Figure 5). Ants, however, do not just use a single training run but will continue to develop their knowledge of the surroundings during multiple runs. We therefore investigated the effect on performance if multiple subtly different training routes were combined. The path integration algorithm that we used allowed the generation of multiple paths that were similar but not identical. The views collected along a number of paths were used to train the network. The learning scheme did not need to be altered as each view collected was simply presented to the network in the order that it was experienced.

**Figure 6:**
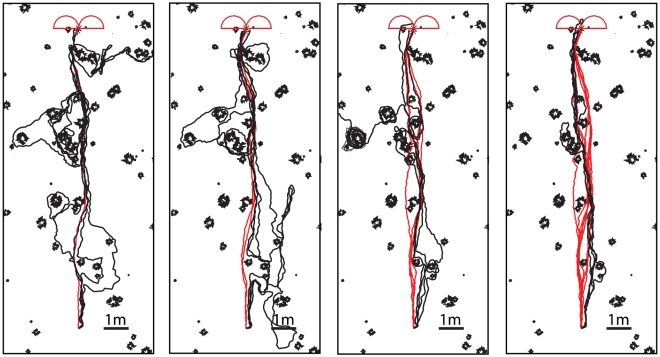
Route following improves with experience. Performance improves as more training runs are performed. Performance is shown following one, two, four and eight training runs. In each figure the training runs used for learning are shown in red while the attempts to recapitulate the route are shown in black. As previously, noise is added to paths during route recapitulation. Of the 4 attempts (black lines) shown in each panel 2, 4, 3 and 4 were successful after one, two, four and eight runs respectively.

Performance is shown for a twelve metre route in one of the more challenging environments (tussock density 0.75 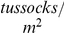) following 1, 2, 4 and 8 training runs (Figure 6). Using multiple training runs can be seen to aid robustness, and after 8 training runs (Figure 6, far right) the recapitulated routes are efficient and consistent, even in this high tussock density environment. With repeated training runs the network will be exposed to a more comprehensive set of views from the route than with a single training run. It should be noted that using, say, four training runs is not the same as sampling the views four times as often during a single training run. In the latter case, sets of four consecutive points are not independent of each other. Using multiple runs however, views from similar locations are coupled only through the environment and thus variation in the views reflects the variation that will be experienced during navigation. For instance, if the distribution of objects in the world means that the training routes are canalised down a narrow corridor, it is likely that the navigated route will also go down a narrow path and so it does not matter that the training views from each run are similar. However, if the route corridor is broader, or even allows multiple paths, then multiple training routes allow a wider set of views that might be experienced when navigating, to be captured. Multiple runs therefore allow a broader, more robust, route corridor to be learnt.

### Learning multiple routes

It has been shown that *Melophorus bagoti* are able to learn and maintain more than one route memory when forced to learn distinct return paths to their nest from a series of different feeders [6]. In the experiments performed by Sommer et al. [6], seven training runs along a first route were followed by a control run to test whether the ant had learnt the route. This training schedule was repeated for a further two routes that each led back to the same location - the nest. Finally, the ants were tested on the first two routes to see if they had retained the original route memories. Here we attempt to replicate this experiment using our route learning algorithm to learn three 10 m routes performed in an environment with a tussock density of 0.75 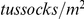.

**Figure 7:**
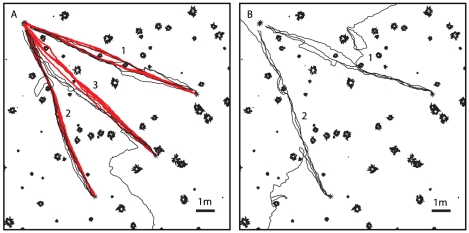
Learning multiple routes. A) Route recapitulation performance (black lines) for each of three routes (red lines) that are learned with the same network. Testing of each of the routes is performed immediately following training on that route and prior to any subsequent learning. The order in which the routes were learnt is indicated by the numbers next to the training routes. B) Performance on the first two routes following learning of all routes, indicating that the route knowledge gained during the first two phases of learning is retained. Having learnt all 3 routes the network must encode 30 m of route information. This increases the likelihood of visual aliasing as is evidenced by the failed recapitulations following learning of all three routes.

To do this we train a network using the first route. The network is then tested before we continue to train the network using views from the second learning route. The network is then again tested before the final training session using views from the third route, before finally being tested on all three routes. The performance can be seen in Figure 7. The network is able to learn and navigate multiple routes without *forgetting* the earlier ones. It is interesting to note that when the third route is recapitulated following learning, the paths tend to get drawn back onto the previously learnt route 2, representing a possible confabulation of these two memories within the network. The individual route memories are not held separately and the return paths for route 3 are drawn back to route 2 as, at that point in the world, views from routes 2 and 3 are similar. This is not wrong *per se*, as the important thing is that the routes lead safely back to the nest. Also this property of routes can be seen in the original paper [6].

## Discussion

We have presented a parsimonious model of visual route guidance which replicates the properties and characteristics of ant navigation. We believe this model represents the only detailed and complete model of insect route guidance to date. However, for us, the major value of the model lies in it being a proof of concept that simple architectures and mechanisms can underpin complex cognitive behaviours such as visually guided routes. Visual navigation requires a cognitive toolkit capable of learning appropriate information, organising memories robustly and also a way of converting those memories into spatial decisions. By considering the way that sensory and motor systems are tightly coupled through behaviour, and utilising familiarity measures to drive route recapitulation, we have produced a minimal cognitive architecture that demonstrates visual route guidance and visually guided search for a goal.

There are three key aspects of this work that we would like to discuss further: (i) Using a familiarity measure to guide routes; (ii) the holistic nature of the route representation; (iii) how learning walks allow route following and nest search with the same mechanism.

### View familiarity and recognition vs. recall

Our own experience tells us that the human capacity for visual recognition is remarkable and clearly outstrips our capacity for recall. For instance, our ability to decide whether we have met somebody before, runs to many more people than those we can explicitly recall specific facts about. Theoretical investigations of abstract neural network models back up this intuition, with familiarity discrimination or recognition models having far greater capacity than associative models with the same number of processing units and weights [60]. Given the limited neural resources available to an ant and the need for rapid learning it makes sense to develop a navigational strategy that relies on recognition, as building either a cognitive map or employing some other form of associative learning are both harder tasks.

The fact that, in our experiments, sensible behaviour can be generated following a single traversal of a route indicates that a form of recognition memory may be sufficient for route navigation in the real world. In fact we would expect that in many ways the problem would be easier for an ant operating in the real world where there would be more information available to disambiguate different views and thereby reduce visual aliasing. The current model presupposes that the only information available to guide behaviour is provided by the high contrast silhouettes of objects against the sky. While we know that ants are able to use skylines to orient themselves [7], any additional visual information, for example colour, texture or celestial cues, information from other modalities [57], [61], [62], or internal motivational cues, would only help to reduce aliasing and improve reliability.

Whether insects have the appropriate brain architecture for storing visual information in this way is not known, though the mushroom bodies would be the obvious candidate neural structure. These higher brain centres, that are enlarged and elaborated in central place foraging insects, have been implicated in a number of cognitive functions including olfactory processing and associative learning [63]–[65], attention [66], sensory integration , sensory filtering [67], [68] and spatial learning [69], [70]. Farris and Schulmeister [71] present compelling evidence that large mushroom bodies receiving visual input are associated with a behavioural ecology that relies heavily on spatial learning. Furthermore, recent research by Stieb et al. [72] implicates the mushroom bodies in the behavioural transition from working inside the nest to foraging outside. In light of our model it would be interesting to evaluate the potential of the mushroom bodies for familiarity discrimination or recognition memory.

The pseudocolour plots in Figure 3 indicate how familiarity could provide another source of information for making routes more robust. If an agent was able to follow a combination of the gradient of the familiarity and the heading of the most familiar direction this would have the effect of drawing the recapitulated paths back onto the habitual route. While this gradient is apparent in the plots that are obtained by sampling from a dense grid of points, it is less obvious how an ant might extract this information, since it would be necessary to sample at least three non-collinear points whilst maintaining the most familiar heading. For a flying insect this would be much less of a problem. The familiarity gradient alone will only serve to draw paths back onto the route and will therefore not produce route following behaviour. However, preliminary results indicate that performance is more robust when the direction indicated by the most familiar view is combined with the familiarity gradient.

We have shown how a familiarity metric could in principle be used to guide successful route navigation; the proposed motor program is however not realistic. Although ants have been observed performing scanning behaviours such as we have used, in general they proceed in a far more purposeful manner when on or near their habitual routes. One issue that we need to address therefore is how familiarity of views could be used in a way that is more consistent with the fine-grained movements that ants actually perform. In order to do this we will need to simulate an environment in which behavioural experiments have been conducted and record in fine detail the movements of ants during their foraging career.

### Holistic representations of visual knowledge

In our second set of experiments we train a network with the views experienced during a learning walk and along a route. There is no requirement for specific views to be selected and following training the network provides a holistic representation of visual information rather than a set of discrete views. The network in fact holds a holistic representation of all the visual information needed for the agent to get to a particular goal, as shown by our replication of multiple route learning. We have previously shown that other neural network models are also able to holistically encode this information [55], [73]. However the particular elegance of the Infomax procedure is that each view is presented to the network once and then discarded.

The consensus view amongst biologists is that ants do not hold spatial information in a unitary cognitive map [12], [74]–[76]. Indeed experiments have shown that the memories required to get to one goal (e.g. the nest) are insulated from the memories required to get to a second goal (e.g. a regular feeding site) [4], [77]. Indeed, if food-bound and nest-ward routes do not overlap then ants captured as they try to get to their nest are effectively lost if they are placed on their familiar food-bound route [4]. Our model could account for this if the motivational state of the animal formed part of the input to the familiarity network. In this way, views would appear familiar only within the correct motivational context.

### Learning walks and behavioural modulation of learning

One of the key properties of this model is that route guidance and place search come from the same mechanism. This comes from incorporating the views experienced during a learning walk into the overall task. Learning walks (and flights in bees and wasps) are a form of active vision where the insect shapes its own perception in a way that is beneficial for learning. This principle is demonstrated by our design of an artificial learning walk. If the views on the outbound sections of the learning walk are made to be more variable than those on the inbound sections, then the inbound views will be learned preferentially. A simple way to achieve this is to have curved outbound routes and straight inbound routes (see the Materials and Methods), a learning walk scheme that performed well. We imagine that when we have an understanding of how real learning walks are structured by the environment, performance will be improved and search paths will more closely resemble those that have been observed in ants.

Another more complex way to modulate learning would be to turn-off learning when not heading towards the nest. This would require some sort of input from the PI system and interestingly, recent detailed descriptions of learning walks in *Ocymyrmex* [34] highlight that PI is likely to be used to ensure ants look at the nest at discrete points during their learning walks. However, these learning walks are still compatible with either behavioural or cognitive modulation of learning. The use of PI might only be used to structure the learning walks and allow the ant to accurately face its nest thereby facilitating behavioural modulation of learning [52].

### Conclusion

We have presented a parsimonious model of visual navigation that uses the interaction of sensori-motor constraints with a holistic route memory, to drive visual navigation. The model captures many of the observed properties of ant navigation and importantly visual navigation is independent of odometric or compass information. Additionally, in the model one does not need to specify when or what to learn, nor separate routes into sequences of waypoints, thus the model is a proof of concept that navigation in complex visual environments can be achieved without those processes. Our principal goal in this research project is to understand the likely and viable mechanisms underpinning insect navigation. Therefore our next step will be to evaluate the model using fine-grained recordings of ants learning and performing routes in their natural habitat.

## Materials and Methods

### The simulation environment

**Figure 8:**
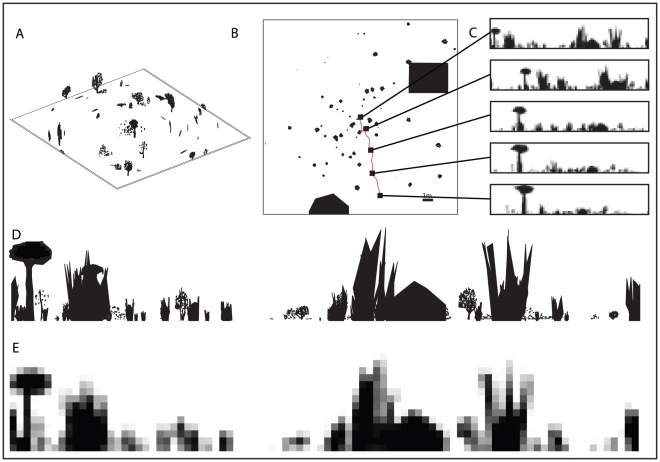
The simulation environment. A,B) Two views of a typical simulated environment used in our experiments. In B the small squares indicate the positions from which the views that are shown in C are taken. C) Five example views taken approximately 2 m apart along a typical route used for learning. The views are oriented so that North (straight up in B) is at the centre of the unwrapped images. D) Typical high-resolution view of the world from an ant's perspective. E) Low-resolution representation of the view shown in D.

To create the environments used in our experiments, a distant panorama of trees and bushes was generated and uniformly distributed densities of tussock-like objects were created over a central 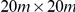 region. While the placement of the tussocks was performed by sampling from a uniform distribution, environments that did not contain many tussocks in the vicinity of the training paths were rejected. In some of the experiments additional 3D objects such as large trees and a building were added within the central region. The environment is intended to produce panoramic views that are typical of the natural environment of the Australian desert ant *Melophorus bagoti* (See [7], [8], [37], [78] for example images of this environment). Figure 8 shows an overview of a typical environment together with a series of views along a route. Notice how variable the views are and also how insignificant the large tree and the building (the solid black objects in Figure 8B) can be from the perspective of an ant. This is easiest to see in Figures 8D and E taken from the middle section of the route where the house, which is NE of the ant (i.e. just over half way along the image; notice the triangular roof in the high-resolution image) blends in to a tussock.

The simulated environment, programmed in MATLAB, consists of objects formed from flat black triangular patches as described below and rendered at a high resolution (Figure 8D), prior to being re-sampled at the low resolution of the simulated visual system (Figure 8C,E). This allows for subtle changes to be registered in response to small movements as would be the case for an ant with a low resolution visual system acting in the real world. This means the resultant view is composed of grey-scale values when a pixel is neither completely covered by sky nor completely covered by an object. In our simulated environment nearby objects are rendered in three dimensions whereas objects at a distance greater than 20 m from the route are flat but oriented so as to be maximally visible.

#### Simulating tussocks, trees and bushes

**Figure 9:**
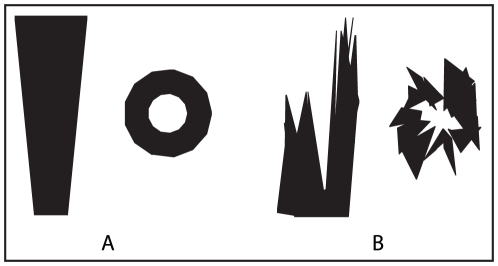
Constructing tussocks from a base model. A) Side and top view of the base model. B) Side and top view of randomly perturbed base model forming a tussock.

Figure 9 shows how the tussocks that form the majority of the objects in the simulated world are constructed. Each tussock is made from a base model consisting of 26 triangular patches that form an inverted cone shape. The vertices of the base model are randomly perturbed and rescaled to produce a variety of similar shaped objects at different scales that approximate the grass tussocks that are typical of the desert ants' natural environment.

**Figure 10:**
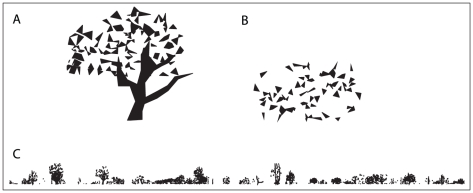
Trees, bushes and the distant panorama. A) Randomly generated tree. B) Randomly generated bush. C) Randomly generated distant panorama.

Figure 10 shows tree and bush objects and a typical panorama, as used in the experiments, prior to being down-sampled to 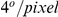. By placing objects sufficiently far away (greater than 20 m), the view of them does not change significantly over the scale of the routes thereby providing a stable frame of reference. The trees and bushes were generated from flat triangular patches oriented so as to be maximally visible from the region of the world where the tussocks and training paths were located. A set of four different base *tree trunks* were used that were randomly flipped and rescaled to provide variation. The leaves of both the bushes and trees were generated from a base template containing a large number of triangular patches. A subset of these patches was randomly chosen for each tree or bush. The environment used in Figures 4 and 5 contained 50 bushes and trees positioned at a uniformly distributed random distance in the range [20–50 m] and random azimuthal angle from the centre of the training route.

### The simulated visual system

Once an environment consisting of triangular patches has been created, a panoramic view from any position within the environment can be generated as follows. We first change the origin of the world to coincide with the position of the simulated ant by subtracting the current x, y and z coordinates of the ant from the set of vertices, X, Y and Z that define the triangular patches. The set of vertices [X, Y, Z] are then converted into spherical coordinates [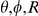] that represent the azimuth, elevation and radial distance. The radial information is discarded and the patches re-plotted in 2D giving the required binary panoramic view (Figure 8D) which is stored as a high-resolution [size 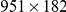] binary matrix. The final step is to reduce the resolution of this image to 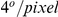, which represents the approximate sampling resolution of the compound eyes of *Melophorus bagoti* workers [78]. The binary matrix is resized to [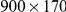] using the *imresize* function in MATLAB and the average value of each [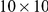] block is then used as the value of the corresponding pixel in the low resolution representation. This averaging results in values in the range [0,1], with values between the two extremes indicating the fraction of sky and objects covered by a pixel in the original high resolution image [Figure 8E].

### Training route generation

The routes shown in Figure 8 and used in the first sets of experiments (reported in Figures 1 and 3) are return paths taken from a paper by Muser et al. [33] that describes the foraging ecology of *Melophorus bagoti*. While we have no knowledge of the real environment from which these paths were recorded we assume that the overall straightness of the paths is somewhat typical and that they therefore represent a reasonable example of the sort of paths that these ants must learn.

**Figure 11:**
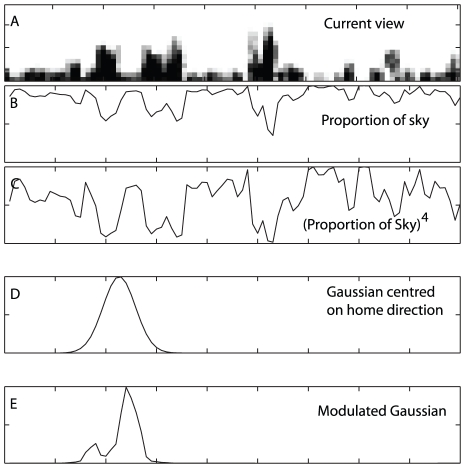
Path integration modulated by obstacle avoidance. A,B,C) Obstacle avoidance is achieved by biasing movements towards low points on the horizon. D) A Gaussian distribution is centred on the home direction. E) The Gaussian is multiplied by the proportion of sky raised to the power of 4 and then normalised. This distribution is then sampled from to determine a movement direction.

In subsequent experiments, paths were generated iteratively starting from the end point of the outbound route using a combination of path integration and obstacle avoidance. Path integration was approximated by centring a Gaussian distribution with a standard deviation of 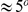 on the correct homeward direction and sampling from this distribution. Obstacle avoidance was incorporated into the path generation scheme by modulating the Gaussian distribution used for path integration by multiplying it by the proportion of sky visible in each direction, 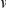 (effectively the inverse of the height of the skyline; Figure 11A,B), raised to the power of 4, 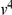 (Figure 11C). The resulting modulated Gaussian (Figure 11E) was renormalized and sampled from to determine a movement direction and a 4 cm step was taken in this direction. Training images are collected after every step. The obstacle avoidance modulation has the effect of biasing movements towards lower portions of the horizon while preventing completely movements towards objects that fill the entire visual field in the vertical direction. Due to the sampling involved in this process, individual paths between two locations will vary slightly allowing the collection of subtly different sets of images describing a route. A return path was considered complete when the distance to the nest was less than 4 cm.

### Learning walks

**Figure 12:**
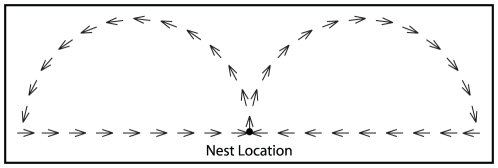
Artificial learning walks. The artificial learning walks are structured so that the outbound sections of the paths are curved while the returns are straight. Behavioural modulation of learning is achieved as views are only consistent during the straight inbound sections.

Where learning walks were added to the training routes, we sampled views from pre-specified paths around the nest. Ants generally walk slower during their learning walks and so samples were taken every 2 cm along the paths as opposed to the 4 cm sampling that was used for generating the route data. These views are added to the start of the set of route views used to train the network in the order that they appear, beginning at the nest. The learning walk in Figure 2B is taken from [33] but see also [34] for another route shape that could have similar properties. The artificial learning walks were generated using a circular path with a radius of 0.5 m for the outbound section and a straight path for the inbound section (Figure 12). This is inspired by data from the learning flights of bumblebees whose early learning flights contain many loops with an inward portion oriented directly at the nest [A. Philippides, personal observation].

### The motor model and scanning routine

**Figure 13:**
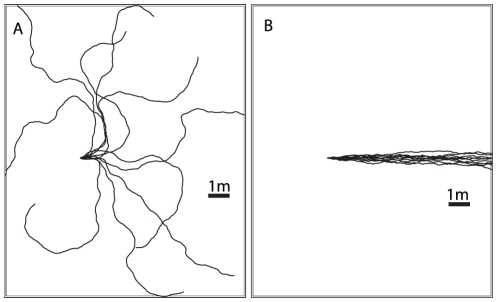
The effects of noise. A) A random walk with normally distributed noise with a standard deviation of 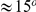 added to the current heading at each timestep and a stepsize of 10 cm. B) A directed walk with a fixed heading of 0 and normally distributed noise with a standard deviation of 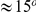 added at each timestep with a stepsize of 10 cm.

In the experiments that we report using a perfect memory system, route recapitulation is performed using a complete 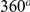 scan of the environment (in steps of 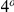) at each timestep. Normally distributed noise with a standard deviation of 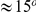 is added to the preferred direction of movement and a 10 cm step is made in this direction (Figure 13).

In the experiments that we report using the Infomax model we employ a slightly more realistic scanning routine during route recapitulation and instead of performing full 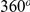 scans we limit the scans to the frontal 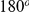 in steps of 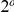 relative to the current heading. We did this to make the scans more similar to those that real ants produce which are rarely as large as 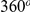. This had a negligible effect on performance except that it made it impossible to follow a path that had any turns greater than 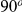 as were present in the Muser et al. learning walk in Figure 2B. As before, normally distributed noise with a standard deviation of 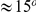 is added to the preferred direction of movement and a 10 cm step is made in this direction. However, when generating the pseudocolor plots in Figures 4 and 5, we did not have a current heading and so performed a full 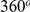 scan to generate an assumed movement direction.

### Navigating with a perfect memory

For the perfect memory system each of the views experienced along a training path was stored. we then calculated a familiarity metric as minus the minimum of the sum squared difference in pixel values between the current view and each of the stored views, 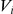.
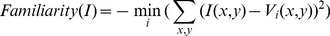
(1)


The maximum familiarity score across all rotated versions of the current view will be obtained for the most similar stored view and the direction from which this maximum was attained determines the next movement to make. In this setting, if the simulated ant does not stray from the training path then it is guaranteed to choose the correct direction to move at each timestep. This is because the most similar view will always be the one that was stored at that location while facing in the direction required to recapitulate the route.

### Familiarity and Infomax

**Figure 14:**
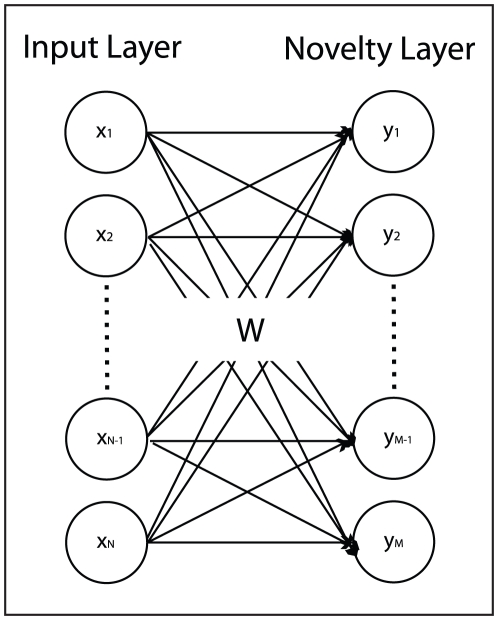
The Infomax model. Circles represent units and arrows denote connections between the input units on the left and the novelty units on the right. There is no output from the network as such since the response of the network is a function of novelty unit activations. Following [59] we therefore do not draw an output layer.

In order to perform familiarity discrimination we chose to use a neural network model that was specifically designed to perform this task [59]. The architecture consists of an input layer and a novelty layer with 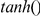 activation functions (Figure 14). The number of input units is equal to the dimensionality of the input which in our case is 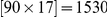, the number of pixels in a down-sampled view of the world. The number of novelty units is arbitrary and here we follow [59] and use the same number of novelty units as inputs. We found that using as few as 200 novelty units can work well in many instances. We did not explore this aspect of the problem in any detail since we were more interested in the behavioural consequences of a familiarity driven approach. The network is fully connected by feedforward connections 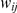. Weights are initialised randomly from a uniform distribution in the range 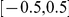 and then normalised so that the mean of the weights feeding into each novelty unit is 0 and the standard deviation is 1. The network is trained using the Infomax principle [79] adjusting the weights so as to maximise the information that the novelty units provide about the input, by following the gradient of the mutual information. The core update equation (4) in our learning scheme performs gradient ascent using the natural gradient [80] of the mutual information over the weights [81] (use of the natural gradient avoids the computationally expensive calculation of the inverse of the entire weight matrix). Since two novelty units that are correlated carry the same information, adjusting weights to maximise information will tend to decorrelate the activities of the novelty units and the algorithm can thus be used to extract independent components from the training data [81]. We choose to use this approach mainly because it only requires a single pass through the data. This means that each view is experienced just once and then discarded. While with a limited amount of data the algorithm is unlikely to converge to a particularly good set of independent components, it is enough that the components that are extracted provide a more suitable decomposition of the training data than of an arbitrary input. During learning the activation of each of the 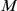 novelty units 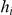 is computed as:
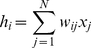
(2)where 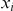 is the value of the 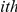 input and 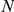 is the number of input units. The output 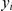 of the novelty units is then given by:
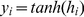
(3)The weights are adjusted using the following learning rule:
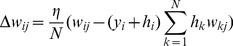
(4)where 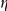 is the learning rate and is set as 0.01 for this paper. Finally, the response of the network to the presentation of an unseen N-dimensional input 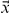 is computed as
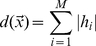
(5)where 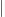 denotes the absolute value. The network response could be viewed as an output layer but as it is a function of the activations of the novelty units, we follow [59] and do not represent it with another layer (Figure 14). As noted above, in this paper we set 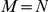 and the network is trained with each training view presented just once to the network in the order in which it is experienced in training. In [59] the authors use 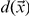 together with a threshold that must be determined empirically to determine whether the input is novel or familiar. For our purposes it is not necessary to determine a threshold as we only need to choose the most familiar input from a limited number of possibilities i.e. the views experienced during a single scan of the environment.

The difference between the way an image difference function and a neural network trained using an Infomax principle represent familiarity will be subtle. In essence, the difference is manifest in the way the information is stored. For image differences, each stored view defines a single point in an n-dimensional space, with n equal to the dimension of the images (n = 90×17 = 1530) and the image difference function gives the squared Euclidean distance of an input image from one of these stored points. This requires all of the views to be stored and so memory load increases as more views are experienced. The Infomax approach instead decomposes each view into a fixed number of components (determined by the number of hidden units in the network) which remains constant, independent of the number of views experienced. The Infomax measure is more abstract and reflects whether a test input is well described in terms of the learned components that the hidden units represent. By decomposing the input in this way it is possible compress redundant data resulting in more efficient memory storage.
